# Biocontrol effect of a solid-state fermentation-derived extract mixture of *Trichoderma asperellum* on sunflower Sclerotinia rot and associated host defense responses

**DOI:** 10.1128/spectrum.04027-25

**Published:** 2026-07-20

**Authors:** Zhilei Chen, Xuesong Li, Minzi Ni, Jing Jin, Jie Chen

**Affiliations:** 1 Zhejiang Green Pesticide 2011 Collaborative Innovation Center, Zhejiang Agriculture and Forestry University 12627 , Hangzhou, China; Connecticut Agricultural Experiment Station, New Haven, Connecticut, USA

**Keywords:** *Trichoderma asperellum *TCS007, derived metabolites, *Sclerotinia sclerotiorum*, antifungal mechanism, plant immune priming

## Abstract

Sclerotinia disease is a destructive fungal disease of sunflowers, soybeans, and other economically important crops, causing substantial yield loss and quality deterioration. Long-term reliance on dose-dependent broad-spectrum fungicides is constrained by resistance risks and potential environmental burdens, creating tension with the sustainability goal of “reducing pesticide use while improving efficacy.” Here, we explore a *Trichoderma* spp.-based microbial disease management strategy. Whole-genome sequencing of *Trichoderma asperellum* TCS007 isolated from Antarctic marine sediments, coupled with genome mining, predicted diverse biosynthetic gene clusters putatively associated with siderophores, polyketides, nonribosomal peptides, and terpenoids; the corresponding metabolites are not chemically confirmed and require further validation. Using a solid-state fermentation workflow, we prepared a fermentation-derived extract mixture (TCS007-SSF-Ex). *In vitro* assays showed dose-dependent inhibition of *Sclerotinia sclerotiorum* by TCS007-SSF-Ex (EC_50_ = 1.252 mg/L), and microscopy revealed cellular damage-consistent changes, including organelle disruption and plasmolysis. Pathogen transcriptomic and metabolism-related analyses indicated broad perturbations in organelle biogenesis and metabolic processes, with significant alterations in pathways associated with succinate, D-glucose, and phenylacetate; these results are consistent with growth inhibition and reduced pathogenicity, but specific molecular targets and causal links remain to be validated. *In vivo*, under certain application conditions, triple applications increased APX activity (+492.5%) and β-1,3-glucanase activity (+419.6%). Collectively, this work supports a “pathogen suppression–host defense induction” framework and facilitates subsequent identification of active components and mechanistic validation.

**IMPORTANCE:**

Sclerotinia diseases cause recurrent and economically important losses in oilseed crops, while long-term fungicide use is constrained by resistance risks and environmental burdens. *Trichoderma*-based biocontrol is a promising complementary strategy, yet evidence supporting metabolite-containing *Trichoderma*-derived preparations as immune elicitors remains less consolidated than that for living inoculants, and scalable production routes are still needed. Here, we examine an Antarctic marine sediment-derived strain, *Trichoderma asperellum* TCS007, and a solid-state fermentation (SSF)-derived extract mixture (TCS007-SSF-Ex) produced via solid-state fermentation. We combine *in vitro* antifungal assays, pathogen ultrastructural observations, and correlative omics analyses with *in vivo* measurements of sunflower defense enzymes (APX and β-1,3-glucanase) to evaluate a “pathogen suppression–host defense induction” framework. Our findings support the potential of SSF-derived *Trichoderma* metabolite mixtures for greener management of Sclerotinia disease and provide a foundation for future chemical identification of active components and mechanistic validation.

## INTRODUCTION

Sclerotinia diseases caused by *Sclerotinia* spp. (1–3), particularly *Sclerotinia sclerotiorum* (4, 5), represent major constraints on sunflower production (6, 7). Infected plants frequently develop head rot (8, 9), stalk breakage (10), and wilting, leading to substantial yield losses (8, 11). Disease management is complicated by the long-term survival of sclerotia in soil (12) and the strong dependence of infection on environmental conditions and crop phenology (13), which together contribute to recurrent outbreaks and inconsistent control outcomes (14).

Current management strategies integrate cultural practices with fungicide-based interventions (15–18). However, chemical control is increasingly constrained by environmental concerns and the emergence of fungicide resistance (19–21). Resistance to succinate dehydrogenase inhibitor (SDHI) fungicides, including boscalid (22–24), has been reported in *S. sclerotiorum* (25–27), and cross-resistance among SDHIs has been documented in multiple cropping systems (25, 28). These limitations underscore the need for diversified disease management strategies that reduce reliance on single-site fungicides and enhance long-term sustainability.

In this context, biological control has gained attention as a complementary and potentially more sustainable approach (29–31). *Trichoderma* spp. are among the most extensively studied fungal biocontrol agents (32–34) and suppress pathogens through multiple mechanisms (35), including nutrient competition (36, 37), antibiosis (38), and mycoparasitism (39, 40). In addition, interactions between *Trichoderma* and host plants may activate or prime defense responses (41), providing an additional layer of protection (42, 43). Genomic analyses further reveal diverse biosynthetic gene clusters and enzyme repertoires putatively associated with secondary metabolite production and cell wall-degrading activities (44, 45), although the corresponding metabolites and functions often require experimental validation.

Despite these advantages, translating *Trichoderma* activity into stable and scalable products remains challenging. Evidence supporting metabolite-containing *Trichoderma*-derived preparations as immune activators is less consolidated than that for living inoculants. Moreover, integrated studies simultaneously examining direct antifungal effects, pathogen cellular and transcriptomic responses, and host defense activation remain limited. Solid-state fermentation (SSF) offers a cost-effective platform for producing biomass and metabolite-associated preparations suitable for scale-up. Here, we investigate an Antarctic marine sediment-derived strain, *Trichoderma asperellum* TCS007, and evaluate an SSF-derived extract mixture (TCS007-SSF-Ex) for (i) antifungal activity against *S. sclerotiorum*, (ii) pathogen ultrastructural and transcriptomic responses consistent with stress and virulence attenuation, and (iii) induction of sunflower defense responses. This integrated assessment provides a framework for subsequent identification of active components and mechanistic validation within sustainable disease management systems.

## MATERIALS AND METHODS

### Tested fungi, plants, and culture conditions

*Trichoderma asperellum* TCS007 was isolated from Antarctic marine sediments by our laboratory. The strain is preserved in the General Microorganism Center of the Chinese Culture Collection of Microorganisms (CGMCC), with the preservation number CGMCC No. 15677. The optimized solid-state fermentation medium contained (46) 25 g wheat bran (Dongsheng, Xuzhou, China), 5 g soybean meal (Macklin, Shanghai, China), 2% (wt/vol) MgSO_4_ (Sinopharm Chemical Reagent, Shanghai, China), 2% (wt/vol) MnSO_4_ (Macklin, Shanghai, China), and 39.7 mL distilled water (initial pH 5.0).

*T. asperellum* TCS007 was prepared by weighing 10 g of the fermentation product in a 250 mL conical flask, followed by the addition of 100 mL of 0.1% Tween water. The mixture was shaken at 28°C and 180 r/min for 1 h. The mycelium and solid medium were filtered through a sterile double gauze cloth to obtain TCS007 spore suspension. The spore concentration was adjusted to 1 × 10^7^ spores/mL by counting with a hemocytometer plate and adding sterile water, then set aside. Cultivation parameters were standardized as follows: 2 mL inoculum (1 × 10⁷ spores/mL), continuous illumination (24 h photoperiod), 28°C incubation temperature, and 15-day culture duration.

*Sclerotinia sclerotiorum* strain, originally isolated from infected sunflower (Zhejiang Province) and preserved by the Zhejiang Collaborative Innovation Center for Green Pesticide (Zhejiang A&F University), was maintained on potato dextrose agar (PDA) medium. Boscalid 50% WG was purchased from Shandong Huimin Zhonglian Biotechnology Co., Ltd.

### Preparation of the SSF-derived extract mixture (TCS007-SSF-Ex)

*T. asperellum* TCS007 was fermented using the solid-state medium and culture conditions described in Tested fungi, plants, and culture conditions (47). The fermented biomass was subjected to triple extraction with ethyl acetate (1:1, vol/vol) at room temperature. After combining the extracts, the solution was vacuum-filtered to remove particulate matter and dehydrated with anhydrous sodium sulfate. The filtered extract was then concentrated under reduced pressure (40°C, 200 mbar) using a rotary evaporator and subsequently reconstituted in dichloromethane. The resulting solution was transferred to sterile centrifuge tubes, where complete solvent evaporation yielded an organic extract mixture, designated as the SSF-derived extract mixture (TCS007-SSF-Ex).

### Whole-genome sequencing of strain TCS007 and prediction of secondary metabolites

Whole-genome sequencing of strain TCS007 was conducted by Meiji Biotechnology Co., Ltd. (Shanghai, China) using an Illumina-PacBio hybrid platform. Raw reads were quality-filtered using Fastp (v0.20.0) and assembled with Hifiasm (v0.19.5), with assembly quality assessed via BUSCO and CEGMA analyses. Gene prediction utilized Maker2 (protein-coding genes), tRNAscan-SE (tRNAs), and Barrnap (rRNAs). Functional annotation was performed against multiple databases (E-value cutoff 1e-5): NR (general functions), Swiss-Prot (curated proteins), Pfam (protein domains), eggNOG (orthologous groups), Gene Ontology (GO) (gene ontology), Kyoto Encyclopedia of Genes and Genomes (KEGG) (metabolic pathways), and CAZy (carbohydrate-active enzymes). Biosynthetic gene clusters (BGCs) were identified using antiSMASH (v8.0.4). Genomic taxonomy was determined through average nucleotide identity (ANI) analysis with OrthoANI, with strains showing <95% ANI similarity considered novel species.

### Assessment of the inhibitory activity against *S. sclerotiorum in vitro*

The sensitivity of *S. sclerotiorum* to TCS007-SSF-Ex was evaluated through a standardized bioassay. First, a 1% (wt/wt) stock solution of TCS007-SSF-Ex in DMF was prepared (nominal concentration: 10,000 mg/L), followed by serial dilution to generate six treatment concentrations (20, 10, 5, 2.5, 1.25, and 0.625 mg/L) in PDA medium. After thoroughly mixing the above six concentrations of drug-containing culture medium, it was poured into a sterile 90 mm diameter petri dish. A PDA plate without any drug was used as the blank control.

Under aseptic conditions, 8 mm mycelial plugs were excised from the colony margins of *S. sclerotiorum* using a sterile punch and inoculated at the center of each treated PDA plate. The plates were incubated at 26°C ± 0.1°C for 72–96 h. When the diameters of colonies in the blank control plate reach 3/4 of the plate’s size, the diameters of the colonies are measured using the cross-crossing method. The average value obtained from two measurements is taken as the colony diameter, and the inhibition rate (*I*) is calculated. This process is repeated three times.


I (%) =[(D₁ − D₂)/(D₁ − 8)] × 100,

where *D*₁ = colony diameter in control plates (mm) and *D*₂ = colony diameter in treated plates (mm). Each treatment was replicated three times.

### Observations of TCS007-SSF-Ex-induced hyphal alterations via scanning electron microscopy and transmission electron microscopy

Mycelial plugs (8 mm diameter) of *S. sclerotiorum* were centrally inoculated onto PDA plates containing 5 mg/L TCS007-SSF-Ex, with untreated mycelia serving as controls (triplicate biological replicates). When the colonies on the control plates occupied roughly three-quarters of the surface area of the Petri dish, the solid medium containing the mycelium from the treated plates was sectioned into pieces measuring 1 × 2 × 1 mm^3^. These pieces were then placed in 2 mL centrifuge tubes containing a 2.5% glutaraldehyde solution and maintained at 4°C for 24 h for fixation. Subsequently, the mycelial pieces were rinsed thrice for 15 min, each with a 0.1 M, pH 7.4 phosphate-buffered saline (PBS) buffer solution. Following primary fixation, the specimens were post-fixed in 1% (wt/vol) osmium tetroxide solution at 4°C for 1 h and then subjected to three consecutive 15-min PBS washing cycles (0.1 M, pH 7.4) to ensure complete removal of residual fixative. The samples were then subjected to a graded ethanol series (30%, 50%, 70%, 80%, 90%, and 95%) for 15 min each for dehydration. After the ethanol was evaporated, the samples were washed twice with 100% ethanol for 20 min each. The samples were adhered to a sample stage with a double-sided conductive adhesive, dried in a sterile environment, sputter-coated with gold, and finally examined under a scanning electron microscope to observe structural changes. Hyphal segments (1 × 2 × 1 mm^3^) from colony margins were fixed in 2.5% glutaraldehyde (4°C for 24 h), rinsed thrice with PBS (0.1 M, pH 7.4; 15 min/wash), and post-fixed with 1% osmium tetroxide (4°C for 1 h). After PBS rinsing (3 × 15 min), samples underwent graded ethanol dehydration (30%–95%, 15 min/step), followed by absolute ethanol treatment (2 × 20 min). Critical point drying and gold sputter coating preceded ultrastructural analysis using a Hitachi SU-8010 SEM (48).

Following dual fixation (as per scanning electron microscopy [SEM] protocol), samples were dehydrated in an ethanol-acetone series (30%–80% ethanol, 15 min/step; 90%–95% acetone, 15 min/step; 2 × 20 min in pure acetone). Epoxy resin embedding, ultrathin sectioning (60–80 nm), and uranyl acetate/lead citrate staining enabled subcellular visualization using a Hitachi H-7650 TEM.

### Transcriptome analysis of *Sclerotiorum* mycelium after TCS007-SSF-Ex treatment

Fresh *Sclerotinia sclerotiorum* mycelia were equally divided into two groups: (i) inoculated into 60 mL of sterile potato dextrose broth (PDB) as the control group (no treatment), and (ii) supplemented with an equivalent mass of mycelia in 60 mL PDB containing 5 mg/L. All cultures were incubated at 28°C with 180 rpm agitation for 48 h. Following this, mycelia were collected by sterile filter paper filtration, rinsed three times with PBS buffer (1 mol/L, pH 7.2), and aliquoted into 1.5 mL microcentrifuge tubes. Samples were flash-frozen in liquid nitrogen, stored at −80°C, and subsequently transported on dry ice to Novogene Co., Ltd. (Beijing, China) for RNA-seq analysis.

#### Sample quality control

RNA integrity was assessed using the RNA Nano 6000 Assay Kit of the Bioanalyzer 2100 system (Agilent Technologies, CA, USA).

#### Library preparation for transcriptome sequencing

Total RNA was used as input material for the RNA sample preparations. Briefly, mRNA was purified from total RNA using poly-T oligo-attached magnetic beads. Fragmentation was carried out using divalent cations under elevated temperature in First-Strand Synthesis Reaction Buffer (5×). First-strand cDNA was synthesized using random hexamer primer and M-MuLV Reverse Transcriptase (RNase H-). Second-strand cDNA synthesis was subsequently performed using DNA Polymerase I and RNase H. Remaining overhangs were converted into blunt ends via exonuclease/polymerase activities. After adenylation of 3′ ends of DNA fragments, adaptors with hairpin loop structure were ligated to prepare for hybridization. In order to select cDNA fragments of preferentially 370–420 bp in length, the library fragments were purified with the AMPure XP system. Then, PCR was performed with Phusion High-Fidelity DNA polymerase, Universal PCR primers, and Index (X) Primer. Finally, PCR products were purified (AMPure XP system), and library quality was assessed on the Agilent Bioanalyzer 2100 system.

#### Clustering and sequencing

The clustering of the index-coded samples was performed on a cBot Cluster Generation System using TruSeq PE Cluster Kit v3-cBot-HS (Illumina) according to the manufacturer’s instructions. After cluster generation, the library preparations were sequenced on an Illumina NovaSeq platform, and 150 bp paired-end reads were generated. Read alignment was performed using HISAT2 (v2.0.5) against the reference genome. Gene expression quantification employed featureCounts (v1.5.0-p3). Differential expression analysis between treatments was conducted with the DESeq2 R package (v1.18.0; Bioconductor, Sydney, NSW, Australia). GO enrichment analysis of differentially expressed genes (DEGs) was implemented through clusterProfiler (v3.8.1). Statistical enrichment of DEGs in KEGG pathways was assessed using KOBAS (v3.0.5; Beijing, China).

### Efficacy evaluation of *T. asperellum* TCS007-SSF-Ex against Sclerotinia rot in sunflowers

Sunflower seeds (cv. “Yunü” oilseed) were surface-sterilized by sequential immersion in 75% (vol/vol) ethanol (1 min) and 1% sodium hypochlorite (5 min), followed by three to four rinses with sterile water. The sterilized seeds were hydroprimed in sterile water at 26°C ± 1°C under dark conditions for 3–4 h and then germinated on sterile filter paper in 9 cm petri dishes (maintained at 26°C in darkness for 24 h).

Germinated seeds were transplanted into pots (95 mm inner diameter × 75 mm height) containing 0.6 kg of mixed substrate (commercial potting soil:field soil = 1:1). The potting soil was sourced from Zhonghe Agricultural Co. (Huaian, China), while field soil was collected from Zhejiang A&F University orchard (Hangzhou, China). Cultivation was conducted in controlled greenhouses at Zhejiang Collaborative Innovation Center for Green Pesticide under the following conditions: 22°C ± 4°C, 50% ± 10% relative humidity, with two seeds sown per pot and thinned to one seedling per pot post-emergence. Plants were maintained under greenhouse conditions following standard cultivation protocols. The experimental design comprised six treatments (Table 1), each containing 15 plants. The ExT1, Ex-T3, and Ex-T5 treatment groups received foliar sprays at 2-day intervals. Twenty-four hours after the final spray application, the first true leaves of sunflower plants were inoculated with *S. sclerotiorum*.

**TABLE 1:** Experimental design for evaluating the biocontrol efficacy of *Trichoderma asperellum* against Sclerotinia rot in sunflower^*a*^

No.	Treatment	Concentration	No. of spray
1	Ex-T1		1
2	Ex-T3	100 mg/L	3
3	Ex-T5		5
4	50% Boscalid W.G.	10 mg/L	1
5	CK (inoculated)	/	1
6	CK (uninoculated)	/	1

^*a*^ For the treatment of sunflower potted plants, Ex-T1, Ex-T3, and Ex-T5 represent spraying once, three times, and five times, respectively. “/” indicates that no concentration was applied for the control treatment. The treatment labeled as 50% Boscalid W.G. refers to spraying with 50% boscalid water-dispersible granules. CK (inoculated) indicates the treatment of spraying with sterile water, while CK (un-inoculated) represents the treatment of spraying with sterile water without subsequent challenge inoculation. The spraying volume for all treatments was 2 mL per pot.

Disease severity rating scale was established as follows: 0, healthy plants with no visible symptoms. 1, brown water-soaked lesions (0–0.8 cm length) on <30% leaves. 3, mild wilting with lesions (0.8–1.6 cm length) affecting 30%–50% foliage. 5, severe blue wilt symptoms, stunted growth, and lesions (1.6–2.4 cm length) on >50% leaves. 7, profuse mycelial growth on stems, significant dwarfing, and lesions (2.4–3.2 cm length). 9, complete plant collapse with black sclerotia in stems and lesions >3.2 cm. Disease severity index (DSI) and disease control efficacy (DCE) were calculated as follows (49):


$$DSI(\%)=100\times \sum_{n=1}^{\infty }\frac{\left(C\times NC\right)}{BC\times NT},$$

$$DCE(\%)=100\times \frac{DSI_{CK}-DSI_{T}}{DSI_{CK}},$$

where *C*, disease class (1, 3, 5…); NC, number of plants in corresponding disease class; BC, the biggest disease class; NT, total number of plants in each treatment; DSI_CK_, disease severity index in CK; and DSI_T_, disease severity index in treatment.

### Immune response activation in sunflowers by *T. asperellum* TCS007-SSF-Ex against Sclerotinia rot

Experiments were conducted using the plants that were subjected to the experimental treatment methods described in Transcriptome analysis of *Sclerotiorum* mycelium after TCS007-SSF-Ex treatment but were not inoculated with *S. sclerotiorum*. Leaves with uniform growth were collected at 48, 72, and 96 h post-treatment (10 leaves/time point), flash-frozen in liquid nitrogen for 5 min, and stored at −80°C for the following experiments (50).

The following biomarkers were quantified using commercial assay kits according to the manufacturer’s instructions (Shanghai Sangon Biotech Co., Ltd., China): reactive oxygen species (ROS): H_₂_O_₂_ and O_₂_⁻; defense-related enzymes: peroxidase (POD), superoxide dismutase (SOD), catalase (CAT), ascorbate peroxidase (APX), phenylalanine ammonia-lyase (PAL), polyphenol oxidase (PPO); and pathogenesis-related proteins: β-1,3-glucanase and chitinase.

### Statistical analysis

Excel 2019 was used to calculate the data. The data were statistically analyzed with SPSS software version 27.0 (IBM Corporation, New York, USA). Visualization was performed using GraphPad Prism software version 10.0 (GraphPad Software, San Diego, CA, USA) and R Studio software. In addition, an analysis of variance with Tukey’s honest significant difference test was conducted to investigate the significant differences between the samples (*P* < 0.05). The number of biological replicates (*n*) for each experiment is specified in the corresponding figure legends.

## RESULTS

### *In vitro* inhibitory activity against *Sclerotinia sclerotiorum*

**Fig 1:**
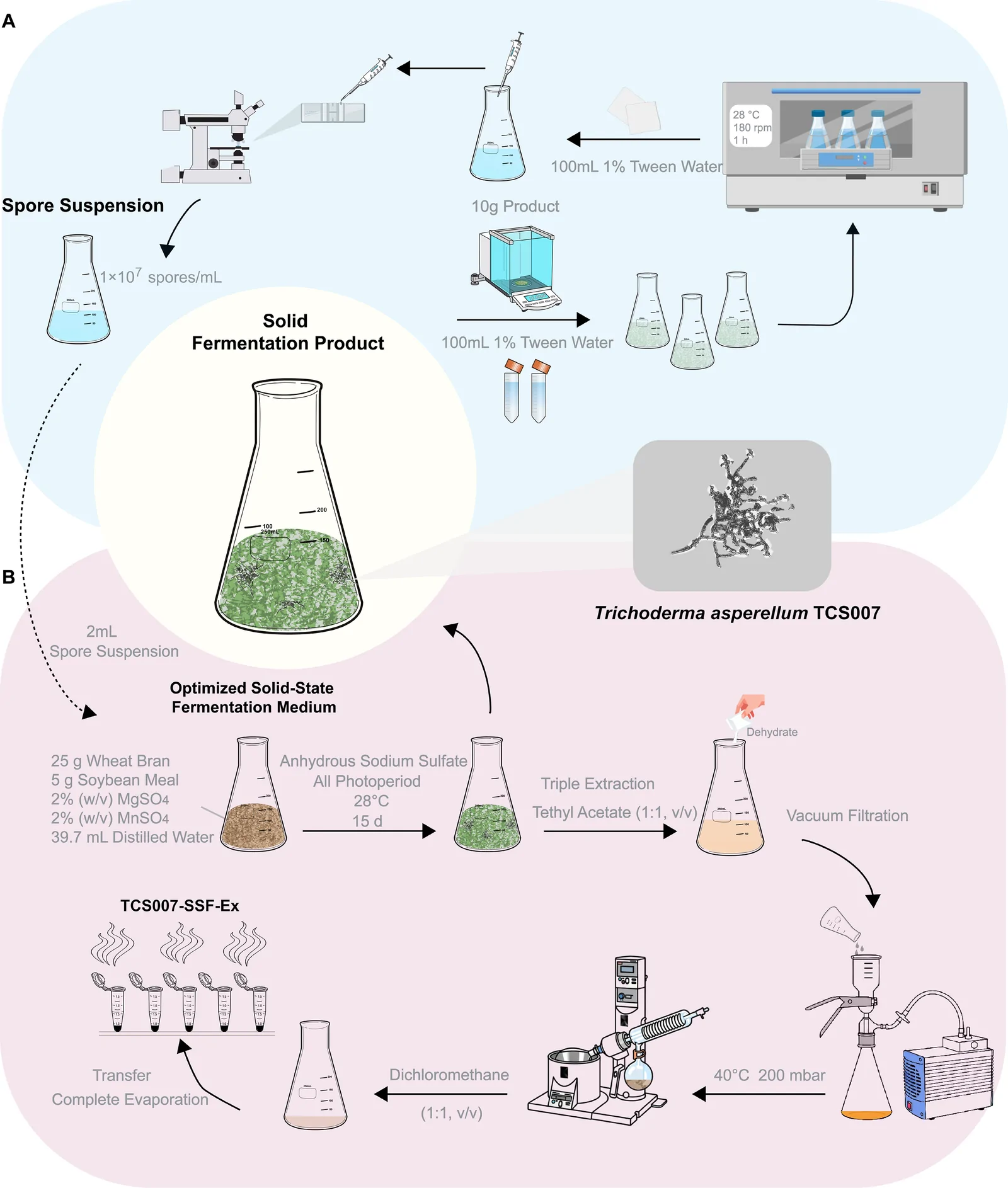
Workflow for preparing the SSF-derived extract mixture (TCS007-SSF-Ex) from *Trichoderma asperellum* TCS007. (**A**) Preparation of spore suspension used exclusively as inoculum for solid-state fermentation. (**B**) Extraction procedure for obtaining the SSF-derived extract mixture (TCS007-SSF-Ex), which was used in all antifungal assays.

The workflow for preparing the SSF-derived extract mixture (TCS007-SSF-Ex) from *T. asperellum* TCS007 is illustrated in Fig. 1. The spore suspension (Fig. 1A) was prepared solely as inoculum for initiating solid-state fermentation and was not used in the antifungal assays. The antifungal activity evaluated in this study was exclusively attributed to the SSF-derived extract mixture (TCS007-SSF-Ex) obtained following fermentation and extraction procedures (Fig. 1B).

**Fig 2:**
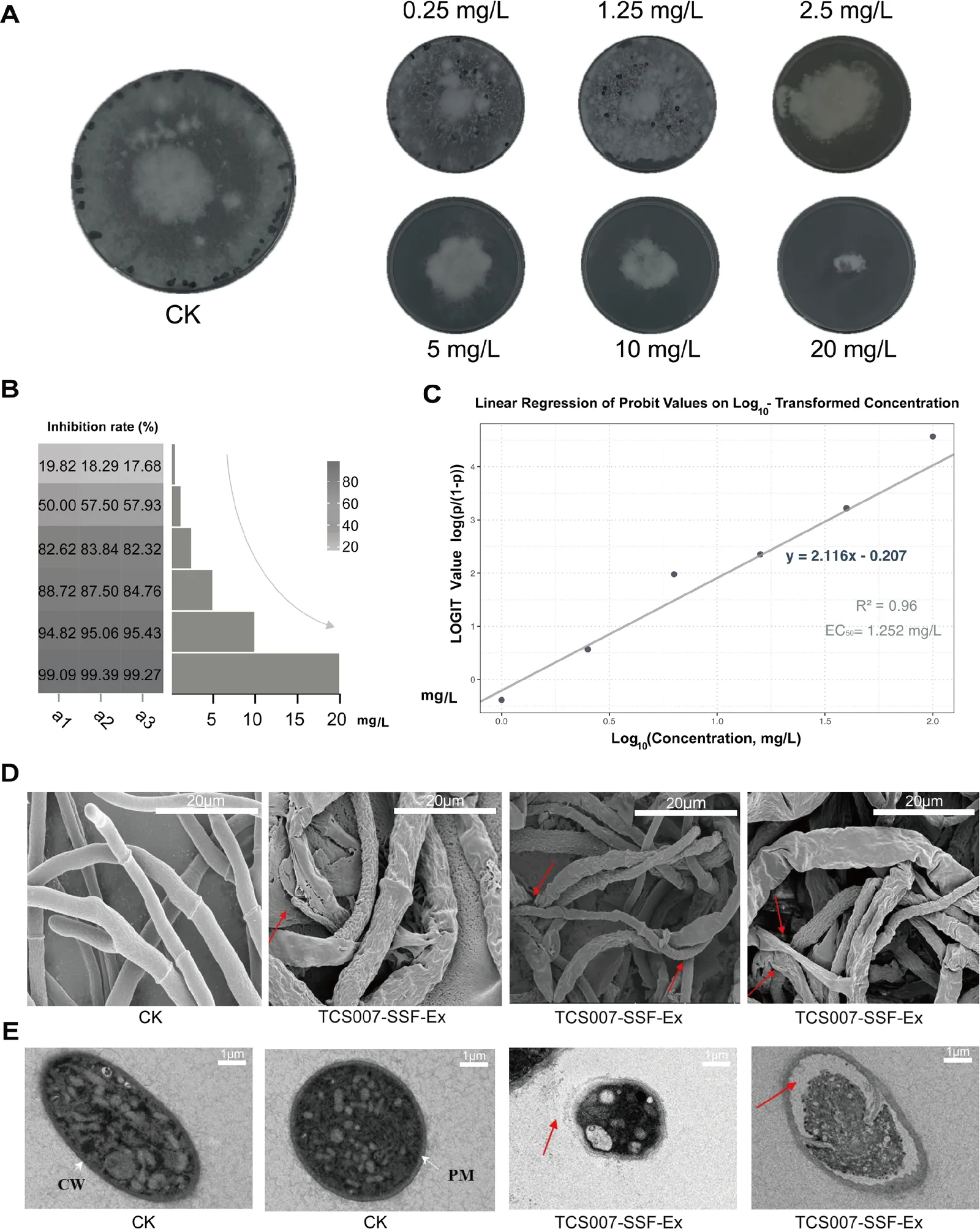
TCS007-SSF-Ex *in vitro* inhibition activity against *Sclerotinia sclerotiorum* and ultrastructural observation. (**A**) Changes in the colonies of phytopathogens on PDA containing TCS007-SSF-Ex at different concentrations after 4-day incubation. (**B**) Inhibition percentage of phytopathogenic colonies treated with different concentrations of TCS007-SSF-Ex. The left part of the picture shows the inhibition rate, while the right part presents the concentration gradient of the experimental design. (**C**) Linear regression of LOGIT values (log[*p*/(1 − *p*]) against the logarithm (base 10) of concentrations (in mg/L). The regression line *y* = 2.116*x* − 0.207 (*R*^2^ = 0.96) describes the relationship, with EC_50_ = 1.252 mg/L, calculated as the concentration at which *p* = 0.5 (*y* = 0 in LOGIT model), indicating 50% effective concentration. (**D**) Scanning electron microscopy analysis revealing morphological alterations. The treatment (5 mg/L) depicted in the figure corresponds to three distinct fields of view obtained via SEM. (**E**) Transmission electron microscopy ultrastructural observations. The treatment illustrated in the figure involves TCS007-SSF-Ex at a concentration of 5 mg/L. CW, cell wall; PM, plasma membrane. Data represent mean ± SD of three independent biological replicates (*n* = 3).

TCS007-SSF-Ex showed dose-dependent inhibition of *S. sclerotiorum* (Fig. 2A), with 20 mg/L causing complete growth arrest (Fig. 2B). The regression model (Fig. 2C) (*y* = 2.116*x* − 0.207, *R*^2^ = 0.96) gave EC_50_ = 1.252 mg/L and EC_90_ = 5.051 mg/L; the high degree of fit (*R*^2^ = 0.96) and EC_50_ value (1.252 mg/L) provide reliable evidence for dose-response relationship analysis.

### Effects of TCS007-SSF-Ex on the ultrastructure of *S. sclerotiorum* hyphae

The three-dimensional spatial structure and ultrastructure of the mycelia of *S. sclerotiorum* were studied by scanning electron microscopy and transmission electron microscopy. In the observation of scanning electron microscopy (Fig. 2D), the diameters of the mycelia in the CK treatment group were uniform, and the surface was smooth and plump without any damage. After treatment with 5 mg/L, the mycelia showed phenomena such as rupture, thinning, and wrinkling, indicating that it has a high-intensity *in vitro* effect. It causes the mycelia to deform by disrupting normal growth and development.

In the transmission electron microscopy observation (Fig. 2E), the ultrastructure of *S. sclerotiorum* in the two CK treatment groups was normal. The cell walls and cell membranes maintained normal morphology, the distribution of organelles in the cytoplasm was uniform, and the contours were clear. After treatment with TCS007-SSF-Ex, the cells lysed, the cell wall contours became blurred, the cell membranes shrank, the electron-transparent areas increased, the protoplasmic vacuolation phenomenon occurred, the intracellular contents decreased, and the organelles dissolved. This indicates that TCS007-SSF-Ex causes severe damage to fungal cellular ultrastructure.

### Details of whole-genome assembly and secondary metabolite prediction of TCS007

**Fig 3:**
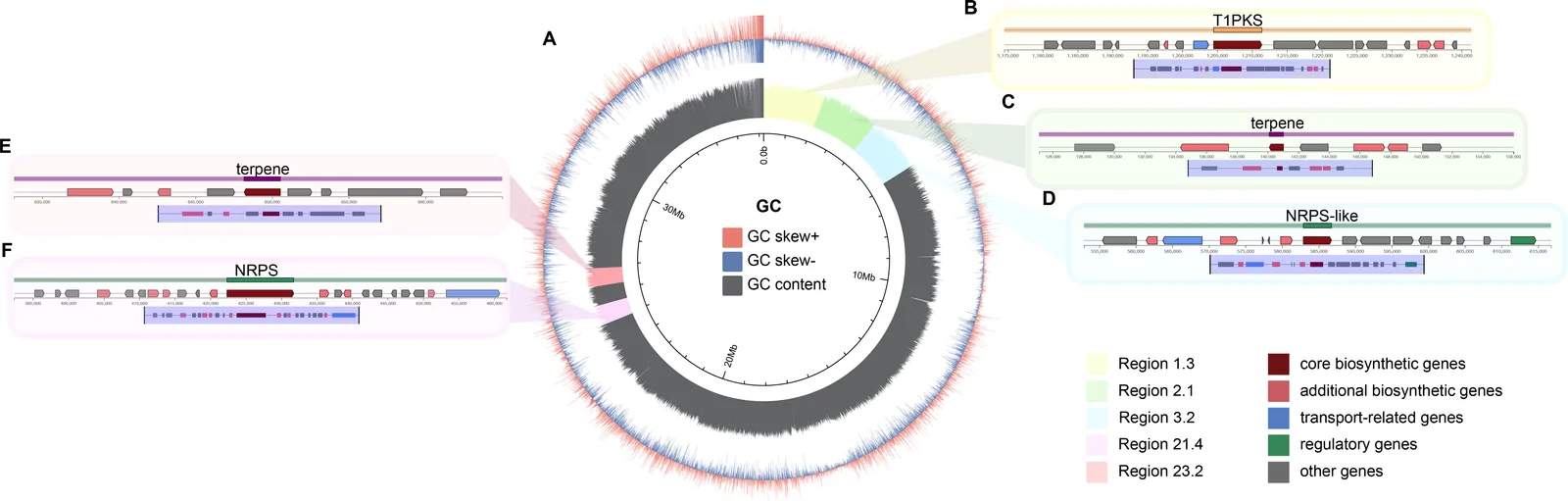
The genomic map of *T. asperellum* TCS007 and the gene cluster for the production of active substances. (**A**) The genomic map of *T. asperellum* TCS007. (**B–F**) The gene clusters (BGC) of the five main active components in the predicted TCS007 strain. Transcriptomic analysis was performed using three biological replicates per treatment (*n* = 3).

The genome of strain TCS007 (Fig. 3A) was sequenced and assembled into 397 scaffolds, with a total length of 36,206,130 base pairs and an average GC content of 48.4%. A total of 10,994 protein-coding genes, 237 tRNA genes, and 50 rRNA genes were predicted.

A comprehensive analysis of the biosynthetic gene clusters of strain TCS007 was conducted using antiSMASH to investigate its secondary metabolite biosynthesis potential. Among the predicted 50 BGCs, 13 showed significant homology to known gene clusters. Only regions with a “High” Similarity Confidence were considered as gene clusters potentially capable of producing compounds with known functions (Table 2). Beyond these, the structures of the secondary metabolites and their corresponding biosynthetic pathways for most BGCs have not been characterized. These findings suggest that strain TCS007 holds potential for the discovery of novel bioactive metabolites and biosynthetic mechanisms.

**TABLE 2:** Secondary metabolite synthesis gene clusters in the TCS007 genome predicted by antiSMASH

Region	Type	Most similar known cluster	Similarity confidence
Region 1.1	NRPS	–^*a*^	–
Region 1.2	NRPS	–	–
Region 1.3	T1PKS	Fusarin C	High
Region 2.1	Terpene	HEx-tc1 terpene	High
Region 2.2	Terpene	Squalestatin S1	Low
Region 2.3	Terpene precursor	–	–
Region 3.1	NRPS-like, T1PKS, NRPS	Fusaric acid	Low
Region 3.2	NRPS-like	Choline	High
Region 4.1	Terpene	–	–
Region 4.2	NRPS	–	–
Region 5.1	Terpene precursor	–	–
Region 5.2	T1PKS	–	–
Region 6.1	Terpene	–	–
Region 6.2	T1PKS	–	–
Region 6.3	T1PKS	–	–
Region 7.1	NRPS-like	–	–
Region 7.2	Isocyanide-nrp	–	–
Region 11.1	Isocyanide-nrp	–	–
Region 11.2	Isocyanide-nrp, isocyanide, terpene	–	–
Region 11.3	T1PKS	–	–
Region 11.4	Terpene	–	–
Region 11.5	T1PKS	–	–
Region 17.1	NRPS-like	–	–
Region 18.1	T1PKS	Cryptosporioptide A/ cryptosporioptide B/ cryptosporioptide C	Low
Region 18.2	Isocyanide	–	–
Region 18.3	NRPS-like, T1PKS	–	–
Region 19.1	Isocyanide	–	–
Region 19.2	NRP-metallophore, NRPS	–	–
Region 20.1	NRPS-like	–	–
Region 20.2	Terpene	Trichobrasilenol/ xylarenic acid B/ brasilane F/ brasilane E/ brasilane D	Low
Region 21.1	T1PKS, NRPS, NRPS-like	–	–
Region 21.2	NRPS	–	–
Region 21.3	T1PKS, NRPS-like, NRPS	Ascochlorin	Low
Region 21.4	Terpene	Clavaric acid	High
Region 22.1	NRPS, T1PKS, terpene	–	–
Region 23.1	Terpene	–	–
Region 23.2	NRPS	Enniatin	High
Region 24.1	T1PKS	–	–
Region 24.2	NRPS-like	–	–
Region 28.1	T1PKS	Aurofusarin	Low
Region 28.2	T1PKS	–	–
Region 30.1	Terpene	–	–
Region 30.2	Terpene, NRPS-like	–	–
Region 37.1	NRPS, T1PKS	–	–
Region 38.1	NRPS	Metachelin C/metachelin A/metachelin-CE/metachelin B/dimerumic acid 11-mannoside/dimerumic acid	Medium
Region 44.1	NRPS-like	–	–
Region 44.2	NRPS-like	–	–
Region 46.1	T1PKS	Trichoxide	Medium
Region 56.1	Terpene	–	–
Region 59.1	NRPS, T1PKS	–	–

^*a*^ “–” indicates that no similar known cluster or similarity confidence was predicted by antiSMASH.

The gene clusters (with “High” Similarity Confidence) potentially possessed by TCS007 were visualized (Fig. 3B through F). Their types were T1PKS, terpene, NRPS-like, terpene, and NRPS, and the most similar known clusters were Fusarin C, HEx-tc1 terpene, choline, clavaric acid, and enniatin, respectively. The detection of BGCs with high sequence similarity to those of known compounds (e.g., Fusarin C, clavaric acid, and enniatin) indicates genetic potential for the biosynthesis of related metabolite classes. However, this *in silico* prediction does not confirm the actual production, accumulation, or presence of these specific compounds in the TCS007-SSF-Ex extract. Targeted chemical analyses, such as liquid chromatography–mass spectrometry (LC–MS), gas chromatography–mass spectrometry (GC–MS), and nuclear magnetic resonance (NMR), are required to determine whether these metabolites are produced under the fermentation conditions used in this study. However, the actual presence and contribution of these compounds in TCS007-SSF-Ex require chemical confirmation.

### Assembly and analysis of the mycelial transcriptome of *S. sclerotiorum*

In this sequencing, the transcriptome analysis of six samples was completed, and a total of 39.38 gigabases (Gb) of clean bases were obtained. Each sample had clean bases exceeding 6.04 Gb. The percentage of Q20 bases was above 98.23%, and the percentage of Q30 bases was above 94.58%. The statistical results of the transcriptome data for the six samples are presented in Table S1. The raw reads ranged from 40,696,342 to 47,644,106. After quality assessment and filtering of low-quality reads, the number of clean reads ranged from 40,273,674 to 47,151,658.

### Transcriptomic responses and functional enrichment analysis of *S. sclerotiorum* following TCS007-SSF-Ex treatment

**Fig 4:**
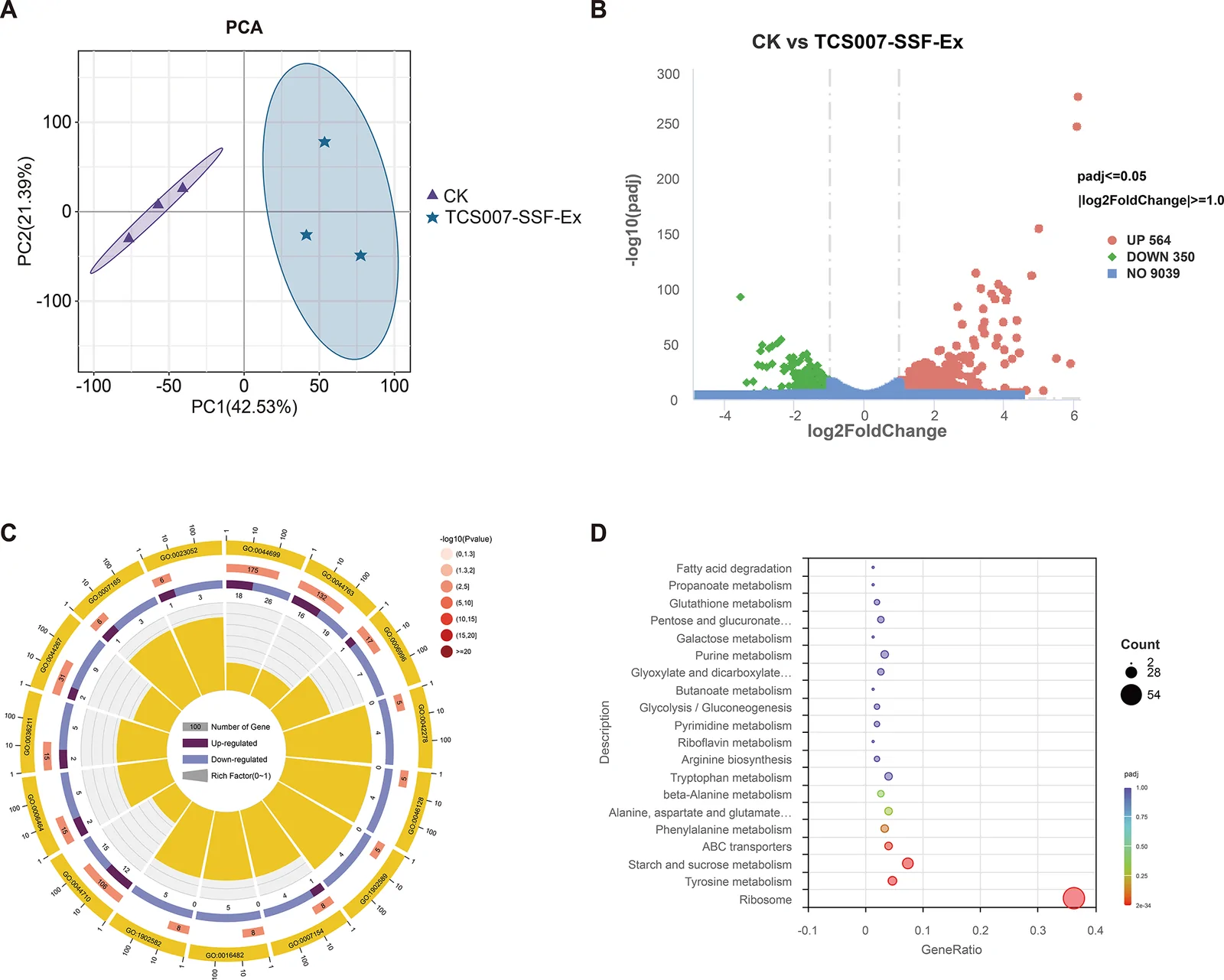
Global transcriptomic responses of *Sclerotinia sclerotiorum* following exposure to the SSF-derived extract mixture (TCS007-SSF-Ex). (**A**) PCA of transcriptomic profiles showing clear separation between control and TCS007-SSF-Ex-treated samples. PC1 and PC2 explain 42.53% and 21.39% of the total variance, respectively (*n* = 3 biological replicates per group). (**B**) Volcano plot showing DEGs between treatment and control conditions. Red dots represent significantly upregulated genes, and green dots represent significantly downregulated genes (|log_2_FC| ≥ 1, adjusted *P* < 0.05). (**C**) Gene Ontology enrichment analysis of DEGs presented as a circular enrichment plot. Functional categories are grouped according to biological processes associated with intracellular organization and transport. (**D**) KEGG pathway enrichment analysis of DEGs. Each dot represents an enriched pathway; dot size indicates the number of DEGs mapped to the pathway, and color intensity corresponds to enrichment significance (adjusted *P*-value).

Transcriptomic profiling revealed substantial transcriptional reprogramming in *S. sclerotiorum* following exposure to TCS007-SSF-Ex. Principal component analysis (PCA) showed clear separation between control and treatment groups, indicating pronounced global transcriptional divergence between the two conditions (Fig. 4A). Differential expression analysis identified 914 differentially expressed genes, including 564 upregulated and 350 downregulated genes (|log₂FC| ≥ 1, adjusted *P* < 0.05) (Fig. 4B). These results indicate that the SSF-derived extract mixture induced widespread transcriptional responses in the pathogen rather than affecting only a limited set of genes.

Gene Ontology enrichment analysis further indicated that many DEGs were associated with intracellular structural organization and cellular transport processes (Fig. 4C). Notably, GO terms such as organelle organization (GO:0006996) and intracellular transport (GO:1902582) were significantly enriched, with single-organism organelle organization (GO:1902589; *q* < 0.001) representing the most statistically significant term. These enrichment patterns are consistent with the ultrastructural alterations observed by transmission electron microscopy—namely mitochondrial cristae disorganization, vacuolar membrane disruption, and nuclear envelope deformation—suggesting that exposure to the extract mixture is associated with destabilization of cellular architecture.

**Fig 5:**
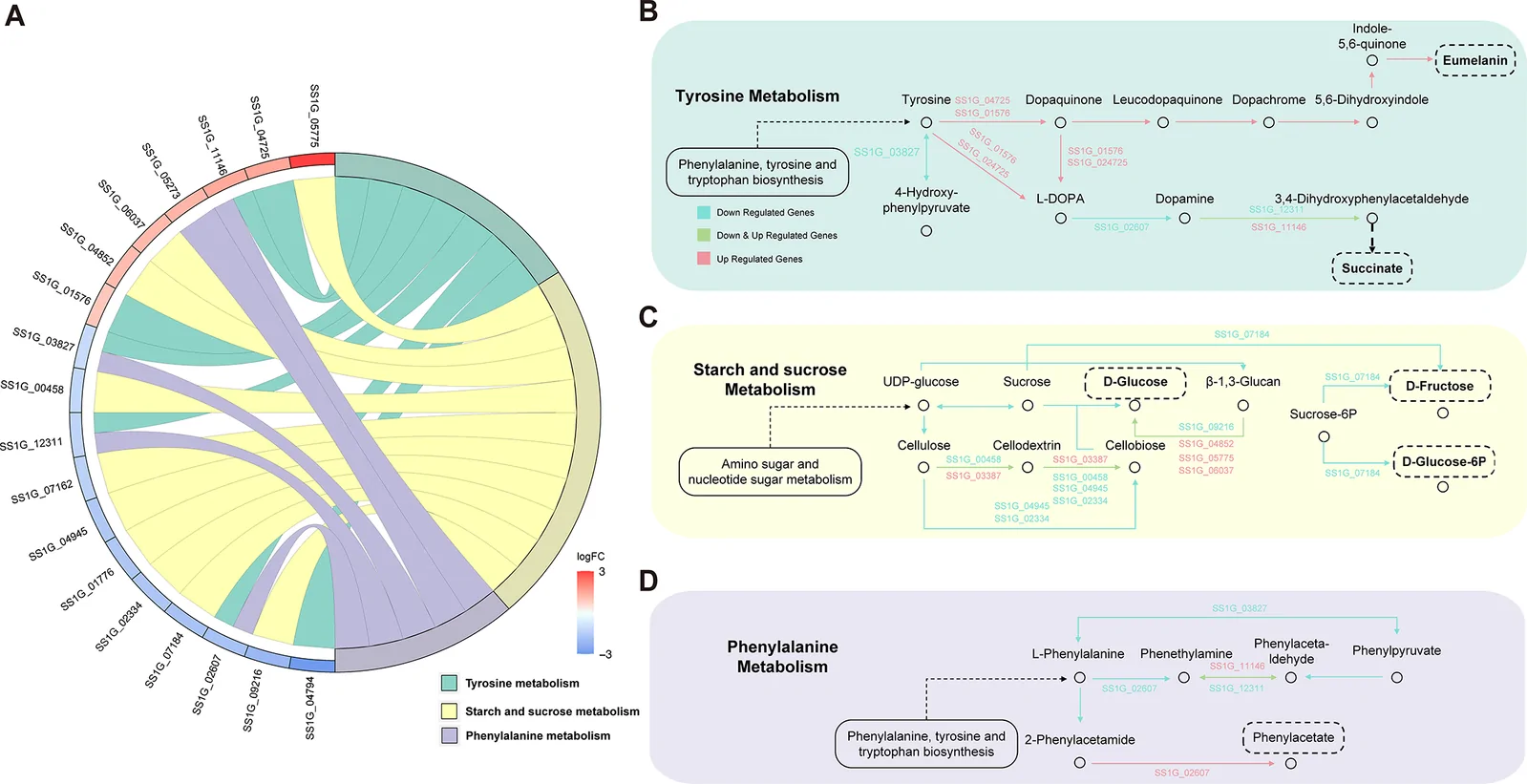
Transcriptional perturbation of three key metabolic pathways in *S. sclerotiorum* following exposure to TCS007-SSF-Ex. (**A**) Chord diagram illustrating regulatory relationships among differentially expressed genes associated with three significantly enriched metabolic pathways identified by KEGG analysis. Colored sectors represent tyrosine metabolism (turquoise), starch and sucrose metabolism (yellow), and phenylalanine metabolism (purple). Chords connect genes involved in these pathways, with color indicating log_2_ fold-change values. (**B–D**) KEGG pathway maps showing transcriptional regulation of genes and associated metabolic reactions within each pathway: (**B**) tyrosine metabolism, (**C**) starch and sucrose metabolism, and (**D**) phenylalanine metabolism. Differentially expressed genes are indicated by color (red, upregulated; blue, downregulated).

KEGG pathway enrichment analysis further highlighted several metabolic pathways significantly affected following TCS007-SSF-Ex treatment (Fig. 4D). Among these, pathways associated with central carbon metabolism exhibited prominent transcriptional modulation. Differential regulation of genes linked to starch and sucrose metabolism, together with changes involving key metabolites such as D-glucose, D-fructose, D-glucose-6-phosphate, and the TCA cycle intermediate succinate, suggests altered cellular energy flux and mitochondrial metabolic activity. The regulatory relationships among three representative pathways—tyrosine metabolism, starch and sucrose metabolism, and phenylalanine metabolism—were further visualized using a chord diagram (Fig. 5A). Because sustained hyphal growth depends on tightly regulated ATP production and biosynthetic capacity, such coordinated metabolic perturbation may contribute to the pronounced growth inhibition observed *in vitro*. Importantly, the simultaneous modulation of multiple metabolic nodes is interpreted here as a signature of systemic cellular stress induced by a complex metabolite mixture rather than evidence for inhibition of specific enzymes by individual compounds.

Detailed pathway mapping further illustrated transcriptional regulation within each pathway, including tyrosine metabolism (Fig. 5B), starch and sucrose metabolism (Fig. 5C), and phenylalanine metabolism (Fig. 5D). Transcriptional shifts associated with phenylacetate-related reactions and eumelanin-associated pathways suggest activation of adaptive or stress-mitigating responses. In filamentous fungi, melanin biosynthesis and aromatic compound metabolism are frequently associated with oxidative stress buffering and environmental resilience. The observed modulation of these pathways may therefore reflect an adaptive response of the pathogen attempting to counteract extract-induced cellular stress.

Importantly, these interpretations are derived from global transcriptomic patterns and should not be construed as evidence of direct inhibition of specific enzymes or defined molecular targets by individual constituents of the SSF-derived extract mixture. Instead, the data support a model in which exposure to TCS007-SSF-Ex triggers coordinated transcriptional reprogramming involving structural destabilization, metabolic imbalance, and activation of adaptive pathways. Together, these responses likely compromise fungal cellular integrity and energy homeostasis, thereby limiting growth and potentially reducing pathogenic fitness under the tested conditions.

Future studies integrating metabolite identification, targeted gene validation, and functional assays will be required to establish causal relationships between specific bioactive constituents and the dysregulated pathways identified in this transcriptomic analysis.

### The role of TCS007-SSF-Ex in activating immune function in sunflower

**Fig 6:**
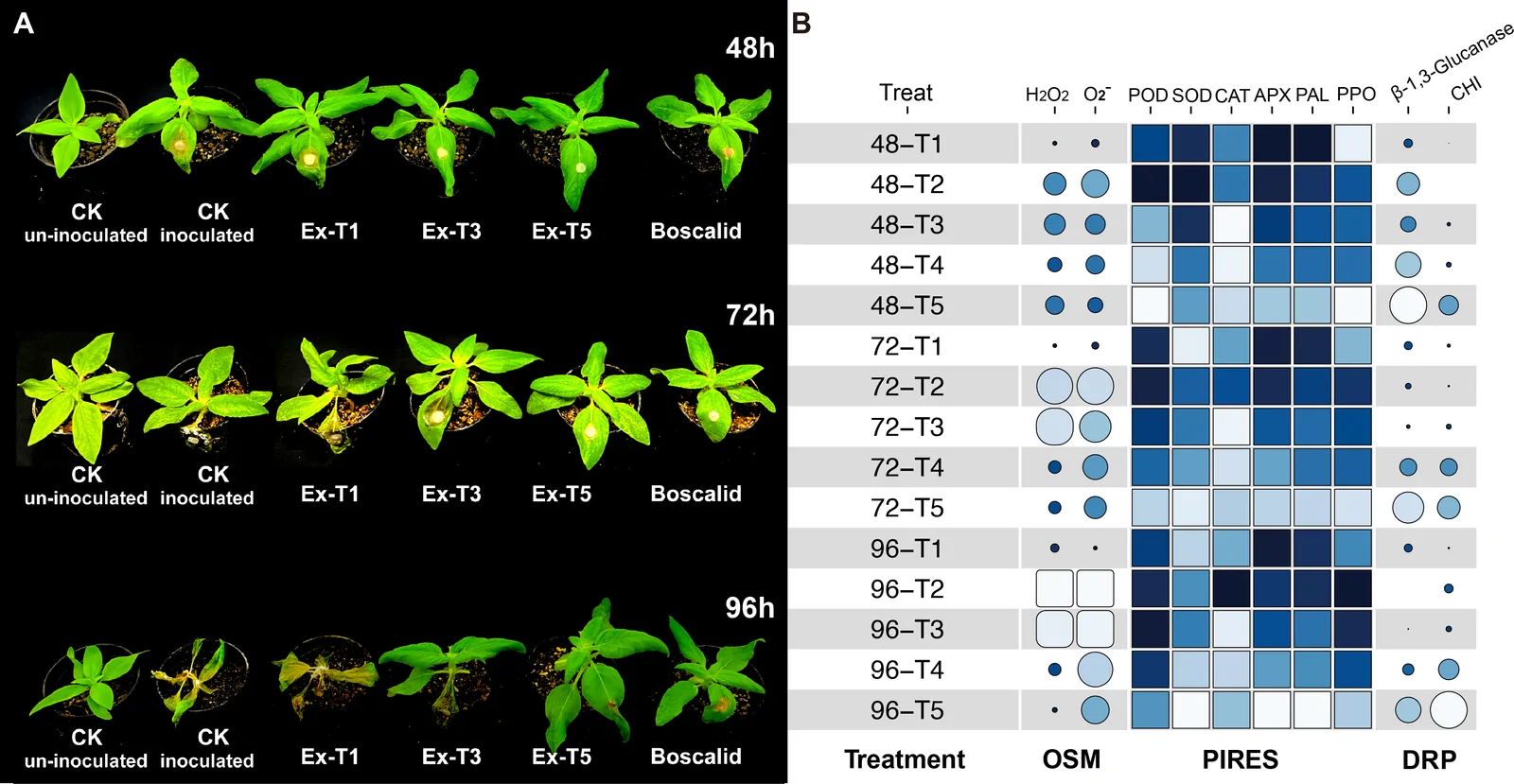
The potential of TCS007-SSF-Ex to activate the immune ability of sunflower and its related physiological and biochemical indexes. (**A**) The incidence of diseases in potted sunflower plants under different treatments at 48, 72, and 96 h after inoculation of pathogenic bacteria. CK (uninoculated); CK (inoculated), 2 mL sterile water was sprayed on each pot. Ex-T1, each pot of plants was sprayed with 2 mL TCS007-SSF-Ex (100 mg/L) once; Ex-T3, each pot of plants was sprayed with 2 mL TCS007-SSF-Ex (100 mg/L) three times; Ex-T5, each pot of plants was sprayed with 2 mL TCS007-SSF-Ex (100 mg/L) five times; 50% Boscalid W.G., 2 mL boscalid (10 mg/L) was sprayed once per pot plant. (**B**) T1, CK (uninoculated); T2, CK (inoculated); T3, Ex-T1; T4, Ex-T3; T5, Ex-T5; OSM, oxidative stress marker; PIREs, plant immune response enzymes; and DRP, defense-related proteins. Disease incidence data were obtained from 15 independent plants per treatment (*n* = 15). Enzyme activity measurements were performed using 10 leaves per time point (*n* = 10). Data are presented as mean ± SD.

Pathogenicity assessments conducted at 96 h post-*S. sclerotiorum* inoculation demonstrated dose-dependent disease suppression (Table 3). Control plants exhibited near-complete infection (99.0% ± 0.13% disease incidence), while TCS007-SSF-Ex treatments showed progressive efficacy enhancement with increased application frequency. The optimal protocol (100 mg/L, five sprays) achieved 58.43% disease incidence reduction (*P* < 0.001) and 59.43% control efficacy (Fig. 6A).

**TABLE 3:** Disease index and defense effectiveness of sunflowers in different treatments^*a*^

No.	Treatment	DSI	DCE
1	Ex-T1	88.84 ± 3.21 b	11.16 ± 3.11 h
2	Ex-T3	58.50 ± 3.28 d	41.50 ± 4.19 e
3	Ex-T5	40.57 ± 0.97 g	59.43 ± 2.90 c
4	50% Boscalid W.G.	19.57 ± 4.22 e	80.43 ± 4.32 a
5	CK (inoculated)	99.0 ± 0.13 a	1.00 ± 0.58 h
6	CK (uninoculated)	/	/

^*a*^ CK (uninoculated) and CK (inoculated) were sprayed with 2 mL sterile water per pot. Ex-T1, Ex-T3, and Ex-T5 indicate that each pot was sprayed with 2 mL of 100 mg/L suspension once, three times, and five times, respectively. For 50% Boscalid W.G., 2 mL of boscalid solution at 10 mg/L was sprayed once per pot. “/” indicates not applicable because no challenge inoculation was performed for the CK (uninoculated) treatment. Data are presented as mean ± SD (n = 15 plants per treatment). Different lowercase letters within the same column indicate significant differences at *P* < 0.05.

Although the disease control efficacy achieved in this study did not reach the level of boscalid, the primary objective was not to demonstrate superior performance relative to chemical fungicides. Instead, TCS007-SSF-Ex should be considered as a complementary strategy within an integrated pest management (IPM) framework. Its potential value lies in reducing chemical input intensity, diversifying selection pressure, and contributing to resistance management, rather than serving as a standalone replacement for high-efficacy fungicides.

### Physiological and biochemical mechanisms of TCS007-SSF-Ex in plant immune activation

The non-inoculated group sprayed with sterile water as the control showed normal plant growth without any disease symptoms. The inoculated group sprayed with sterile water and infected with *S. sclerotiorum* developed severe disease symptoms (Fig. 6A).

Sunflower plants exhibited progressively severe disease symptoms at 48, 72, and 96 h post-inoculation. Physiological analyses demonstrated (Fig. 6B) that POD, SOD, APX, PAL, and PPO enzyme activities significantly increased with additional TCS007-SSF-Ex treatments, while CAT activity slightly decreased, potentially regulated through complementary enzymatic mechanisms to optimize oxidative stress responses. For instance, the enhanced activities of SOD, POD, and APX compensated for reduced CAT activity.

In inoculated controls (CK), H_₂_O_₂_ content significantly increased over time, reaching peak levels at 96 h. By contrast, T1, T3, and Ex-T5 treatments reduced H_₂_O_₂_ accumulation, with Ex-T3 decreasing levels by 17.1%, 37.9%, and 46.4%, and Ex-T5 by 8.4%, 38.4%, and 56.9% at 48, 72, and 96 h, respectively. Superoxide anion (O_₂_−) followed similar suppression patterns.

The treatments demonstrated significant enhancement of plant immune responses and oxidative stress tolerance through dose-dependent activation of antioxidant enzymes and defense-related enzymes in *S. sclerotiorum*-inoculated plants. In contrast to CK inoculated, Ex-T5 (maximum number of induction) showed the most pronounced effects, with APX activity increasing by 492.5% at 48 h and PAL activity rising by 146.8% at 96 h, indicating robust induction of phenolic metabolism and redox homeostasis. Ex-T3 also displayed remarkable performance, particularly in β-1,3-glucanase (186.9% increase at 96 h) and chitinase (89.5% increase at 72 h), which synergistically degrade fungal cell walls and suppress pathogen colonization. These results are in agreement with existing studies showing that β-1,3-glucanase and chitinase can enhance the antifungal activity by synergistically degrading the fungal cell wall (51, 52).

Notably, compared with CK inoculated, the activation patterns of antioxidant enzymes (POD, SOD, CAT, and APX) varied across treatment groups. Ex-T5 induced the highest APX activity (492.5% at 48 h), which efficiently scavenges hydrogen peroxide, while Ex-T1 showed the most prolonged CAT activation (394.5% at 96 h), suggesting distinct roles in oxidative stress management at different infection stages. The sequential activation of PAL (90.0% at 48 h) and PPO (240.0% at 96 h) in Ex-T5 further supports the hypothesis that promotes phenolic compound accumulation, reinforcing cell wall integrity and inhibiting pathogen penetration (53).

In contrast to CK inoculated, the dose-dependent response was evident in β-1,3-glucanase and chitinase activities, where Ex-T5 consistently outperformed T3. For example, β-1,3-glucanase activity in Ex-T5 increased by 419.6% at 96 h, contrasting with 186.9% in T3, indicating stronger induction of plant cell wall-degrading enzymes at higher concentrations. This aligns with the role of these enzymes in disrupting fungal cell wall structure and triggering pathogen-associated molecular pattern-triggered immunity (54, 55).

Collectively, these results demonstrate that treatments enhance plant resistance against *S. sclerotiorum* by activating a multi-layered defense network, including antioxidant systems, phenolic biosynthesis, and cell wall-degrading enzymes. The observed dose-dependent effects suggest that Ex-T5 represents an optimal concentration for balancing immune activation and resource allocation, offering a promising strategy for sustainable management of fungal diseases in agricultural systems. Future studies should investigate the molecular mechanisms underlying mediated immune priming, such as the involvement of MAPK signaling pathways and transcriptional regulation of defense genes.

## DISCUSSION

The biopesticide potential of *Trichoderma* fungi has been extensively explored (33, 56, 57), particularly in plant disease control (58–60). However, current research predominantly focuses on their role as beneficial microorganisms with partial chemical pesticide substitution capabilities (34, 61), while studies investigating their function as immune activators remain limited. This study provides evidence supporting a dual mode of action associated with TCS007-SSF-Ex, combining direct antifungal effects with host defense activation. However, under the tested greenhouse conditions, the maximum disease control efficacy (~59%) was substantially lower than that achieved by the reference fungicide boscalid (~80%), indicating that the extract mixture cannot yet match the performance of conventional high-efficacy chemical fungicides. These findings support the potential of TCS007-derived metabolites to contribute to host defense responses. *Trichoderma* conidia exhibit antagonistic activity, cost-effective fermentation, and rapid proliferation (62, 63).

Comparative analysis of the predicted biosynthetic gene clusters in strain TCS007 suggests that most high-confidence clusters correspond to metabolite classes commonly reported in *Trichoderma* species, including NRPS-, T1PKS-, and terpene-associated clusters. Similar categories of secondary metabolite biosynthetic genes have been documented in previously sequenced *T. asperellum* strains and other *Trichoderma* spp., indicating that TCS007 shares conserved biosynthetic potential within the genus (64–66). Notably, while several clusters exhibited homology to known biosynthetic pathways (e.g., those associated with fusarin C, clavaric acid, and enniatin), the present study did not lead to the identification of any chemically validated novel metabolite. Moreover, the majority of predicted BGCs displayed low or uncharacterized similarity, and their corresponding products remain undefined. Therefore, although the genomic data highlight biosynthetic diversity, conclusions regarding the discovery of new compounds require targeted chemical isolation and structural confirmation.

While repeated applications (five sprays) provided the greatest disease suppression under controlled greenhouse conditions, complete control of Sclerotinia rot was not achieved. The primary objective of this study was to evaluate the intrinsic biocontrol potential of the SSF-derived extract mixture (TCS007-SSF-Ex) rather than to establish an optimized field application program. Translating this biological potential into a cost-effective and practical management strategy will require further development. Future research should therefore focus on formulation improvement, enrichment or identification of active constituents, and dose optimization to enhance efficacy and potentially reduce application frequency. In addition, integration with reduced-rate chemical fungicides or other biological agents within an IPM framework may further improve both disease control performance and economic feasibility.

The antagonistic capacity involves multiple synergistic mechanisms, including nutrient competition and direct parasitism (67–69), mediated through antifungal metabolites and cell wall-degrading enzymes (70–72). These findings provide a foundation for deeper molecular mechanistic studies. The rapid development of bioinformatics technologies, including transcriptomic, metatranscriptomic, mitochondrial genomic, and population genetic approaches, has made it increasingly feasible to investigate complex biological interactions, metabolic adaptation, and host-associated differentiation (73–75). However, it must be acknowledged that in this study, whether in the prediction of genomic data and derived secondary metabolites or the transcriptomics-based exploration of molecular mechanisms, there is a significant lack of extensive validation experiments. We believe this is not an isolated case but a common phenomenon. Therefore, the validation of these related aspects will be a critical next step in our work. Plant cells under pathogen stress experience oxidative stress due to electron leakage to superoxide anion (O_₂_⁻) during photosynthesis and respiration (76), elevating ROS production (76–78). Pathogen infection or abiotic stress significantly increases O_₂_⁻ and H_₂_O_₂_ levels (78–80). Reactive oxygen species directly damage cellular lipids, proteins, and DNA, leading to cellular death (81). The plant antioxidant defense system tightly regulates the balance between ROS production and scavenging (82), with superoxide dismutase, catalase, and peroxidase serving as core plant immune response enzymes in ROS homeostasis (83). Defense-related proteins are typically constitutively expressed at low levels in plants but are significantly upregulated upon fungal, bacterial, or viral pathogen infection. Host assays in this study demonstrated that sunflower immune responses to *S. sclerotiorum* involve prolonged and complex processes. Although TCS007 treatment may be associated with enhanced sunflower defense responses, the underlying priming mechanisms and sustained activation dynamics require further investigation.

These findings provide preliminary evidence that SSF-derived *Trichoderma* metabolite mixtures may function as adjunctive immune-activating agents, contributing to diversified disease management strategies rather than replacing conventional fungicides. These findings may contribute to the development of sustainable disease management strategies in oilseed crop systems.

## Data Availability

The sequencing data associated with this study have been deposited in the Genome Sequence Archive (GSA) under BioProject accession number PRJCA061756. The whole-genome sequencing data of *Trichoderma asperellum* TCS007 are available under GSA accession number CRA042965, and the RNA-seq data of *Sclerotinia sclerotiorum* treated with TCS007-SSF-Ex are available under GSA accession number CRA043008.

## SUPPLEMENTAL MATERIAL

Table S1 spectrum.04027-25-s0001.docx Evaluation statistics of sequencing data.

ASM does not own the copyrights to Supplemental Material that may be linked to, or accessed through, an article. The authors have granted ASM a non-exclusive, world-wide license to publish the Supplemental Material files. Please contact the corresponding author directly for reuse.
