# Supplementary material for: Biocontrol effect of a solid-state fermentation-derived extract mixture of Trichoderma asperellum on sunflower Sclerotinia rot and associated host defense responses
Source: Microbiol Spectr. 2026 Jul 20;14(9):e04027-25. doi: 10.1128/spectrum.04027-25 (PMC13532180; doi:10.1128/spectrum.04027-25)
Supplement: Supplementary file 1 [file spectrum.04027-25-s0001.docx]

Supplementary Material

**Supplementary Tables 1.** Evaluation statistics of sequencing data.

| **sample** | **raw reads** | **raw bases** | **clean reads** | **clean bases** | **error rate** | **Q20** | **Q30** | **GC pct** |
| --- | --- | --- | --- | --- | --- | --- | --- | --- |
| CK-1 | 43737518 | 6.56G | 43328992 | 6.5G | 0.01 | 98.34 | 94.91 | 46.61 |
| CK-2 | 44360234 | 6.65G | 43923492 | 6.59G | 0.01 | 98.38 | 95.03 | 46.58 |
| CK -3 | 44438364 | 6.67G | 43956642 | 6.59G | 0.01 | 98.33 | 94.84 | 46.47 |
| 007-1 | 44374848 | 6.66G | 43909866 | 6.59G | 0.01 | 98.41 | 95.09 | 46.2 |
| 007-2 | 40696342 | 6.1G | 40273674 | 6.04G | 0.01 | 98.23 | 94.58 | 46.11 |
| 007-3 | 47644106 | 7.15G | 47151658 | 7.07G | 0.01 | 98.35 | 94.91 | 46.17 |
